# Draft genome sequence of multidrug-resistant *Citrobacter freundii* MTR_GS_V1777 strain isolated from a spinach (*Spinacia oleracea*) sample in Gazipur, Bangladesh

**DOI:** 10.1128/mra.01082-23

**Published:** 2024-01-11

**Authors:** Md. Saiful Islam, Pritom Kumar Pramanik, Md. Liton Rana, Srinivasan Ramasamy, Pepijn Schreinemachers, Ricardo Oliva, Md. Tanvir Rahman

**Affiliations:** 1 Department of Microbiology and Hygiene, Faculty of Veterinary Science, Bangladesh Agricultural University, Mymensingh, Bangladesh; 2 Department of Animal Sciences, University of California—Davis, Davis, California, USA; 3 World Vegetable Center, Shanhua, Tainan, Taiwan; 4 World Vegetable Center, East and Southeast Asia, Bangkok, Thailand; The University of Arizona, Tucson, Arizona, USA

**Keywords:** vegetable, whole genome, *Citrobacter freundii*, multidrug resistance, virulence, gardening system, Bangladesh

## Abstract

We announce a genome sequence of *Citrobacter freundii* MTR_GS_V1777 strain isolated from a vegetable sample in Bangladesh. This strain had a genome size of 4,997,753 bp (58.7× genome coverage) and contained two plasmids, typed as sequence type ST124, 38 predicted antibiotic resistance genes, and 77 predicted virulence factor genes.

## ANNOUNCEMENT

Vegetables are commonly exposed to microbial contamination due to contact with soil, dust, untreated irrigation water, and handling during harvesting and postharvest processing (1). *Citrobacter* species are among the bacteria that have been found in vegetables (2). The widespread and improper use of antimicrobial agents has led to the rise of antimicrobial resistance and multidrug resistance (MDR) in bacteria, including *Citrobacter* species (3, 4).

Between September 2022 and March 2023, fresh spinach (*Spinacia oleracea*) leaf samples were collected from the surface garden in the Gazipur district of Bangladesh (24.0958°N, 90.4125°E) and brought to the laboratory (24.7245°N, 90.4372°E). The collected samples were processed according to the methods outlined in a previous study (5). The samples were carefully chopped, weighed (50 g), and placed into a sterile polyethene stomacher bag with 200 mL of buffered peptone water. Subsequently, they were macerated for 5 minutes at 230 rpm using a Stomacher 400 circulator (Seward Ltd., London, UK). The processed samples were then incubated at 37°C for 24 hours, spread onto xylose-lysine deoxycholate agar plates, and incubated at 37°C overnight. The resulting colonies were subjected to Gram staining and biochemical tests to isolate *Citrobacter freundii* (6). Identification of *C. freundii* was accomplished using matrix-assisted laser desorption ionization time-of-flight mass spectrometry (7). The disk diffusion method (8) and the Clinical and Laboratory Standards Institute guidelines (9) were applied to ascertain their MDR properties. The MDR *C. freundii* MTR_GS_V1777 isolate, showing phenotypic resistance to gentamicin, streptomycin, ciprofloxacin, erythromycin, azithromycin, cephalexin, tetracycline, ampicillin, cotrimoxazole, and sulfonamide, was selected in this study. A single colony was then picked from the overnight culture in nutrient broth (HiMedia, India) at 37°C, and the DNA was extracted with a DNeasy Blood and Tissue Kit (QIAGEN, Hilden, Germany). Subsequently, a DNA library was prepared using the Nextera DNA Flex Library Prep Kit (Illumina, San Diego, CA, USA). Genome sequencing was performed on the Illumina NextSeq 2000 platform with 2 × 150 bp reads. The genome assembly involved Unicycler v.0.4.9 (10) and included initial raw paired-end read trimming (*n* = 1,215,420) with Trimmomatic v.0.39 (11) (leading: 20, sliding window: 4:20:20, trailing: 20, and minlen = 36) to remove Illumina artifacts and phiX reads. Quality assessment was conducted using FastQC v.0.11.7 (12), and genome annotation was done by PGAP v.6.6 (13). Within the assembled genome, sequence type was predicted using MLST v.2.0 (14); pathogenicity index was obtained using PathogenFinder v.1.1 (15); and plasmids were identified with PlasmidFinder v.2.1 (16). Antibiotic resistance genes (ARGs) were predicted using CARD v.3.2.4 (RGI main) (17), with specific criteria for perfect (100% identity) and strict (>95% identity) matches to curated reference sequences in the CARD databases. Virulence factor genes (VFGs) were predicted through VFDB with VFanalyzer (18), and metabolic functional features were identified with RAST v.2.0 (19). Default settings were used for all software unless otherwise noted.

The genome coverage of the *C. freundii* MTR_GS_V1777 strain was 58.7×, and a total of 53 contigs were obtained. Our assembled genome had a total length of 4,997,753 bp, a guanine-cytosine content of 51.8%, four contig L50, and an N50 value of 514,523 bp. It contained a total of 4,822 genes, 4,740 coding sequences, 4,670 protein-coding sequences, 82 RNA genes, and 70 pseudogenes. This assembled genome predicted two plasmid replicons, i.e., IncFII(K) and IncR. Moreover, this genome consisted of a sequence type of ST124 and a pathogenicity index of 0.875. The strain carried 38 predicted ARGs and 77 predicted VFGs. Moreover, this genome contained 386 subsystems (having 2,259 genes) with 32% coverage.

## Data Availability

The whole-genome sequencing shotgun analysis of *Citrobacter freundii* MTR_GS_V1777 was deposited to GenBank under accession number JAVTVR000000000. The relevant data, including the raw reads, were also submitted with BioProject accession number PRJNA1020260, BioSample accession number SAMN37518445, and SRA accession number SRR26151867. In this paper, the specific version being referred to is identified as JAVTVR000000000.1.
